# Supplementary material for: The Application of Clustering on Principal Components for Nutritional Epidemiology: A Workflow to Derive Dietary Patterns
Source: Nutrients. 2022 Dec 30;15(1):195. doi: 10.3390/nu15010195 (PMC9824338; doi:10.3390/nu15010195)

# Supplementary Materials

**Figure S1**. Heatmap showing factor loadings for each food category and each principal component.

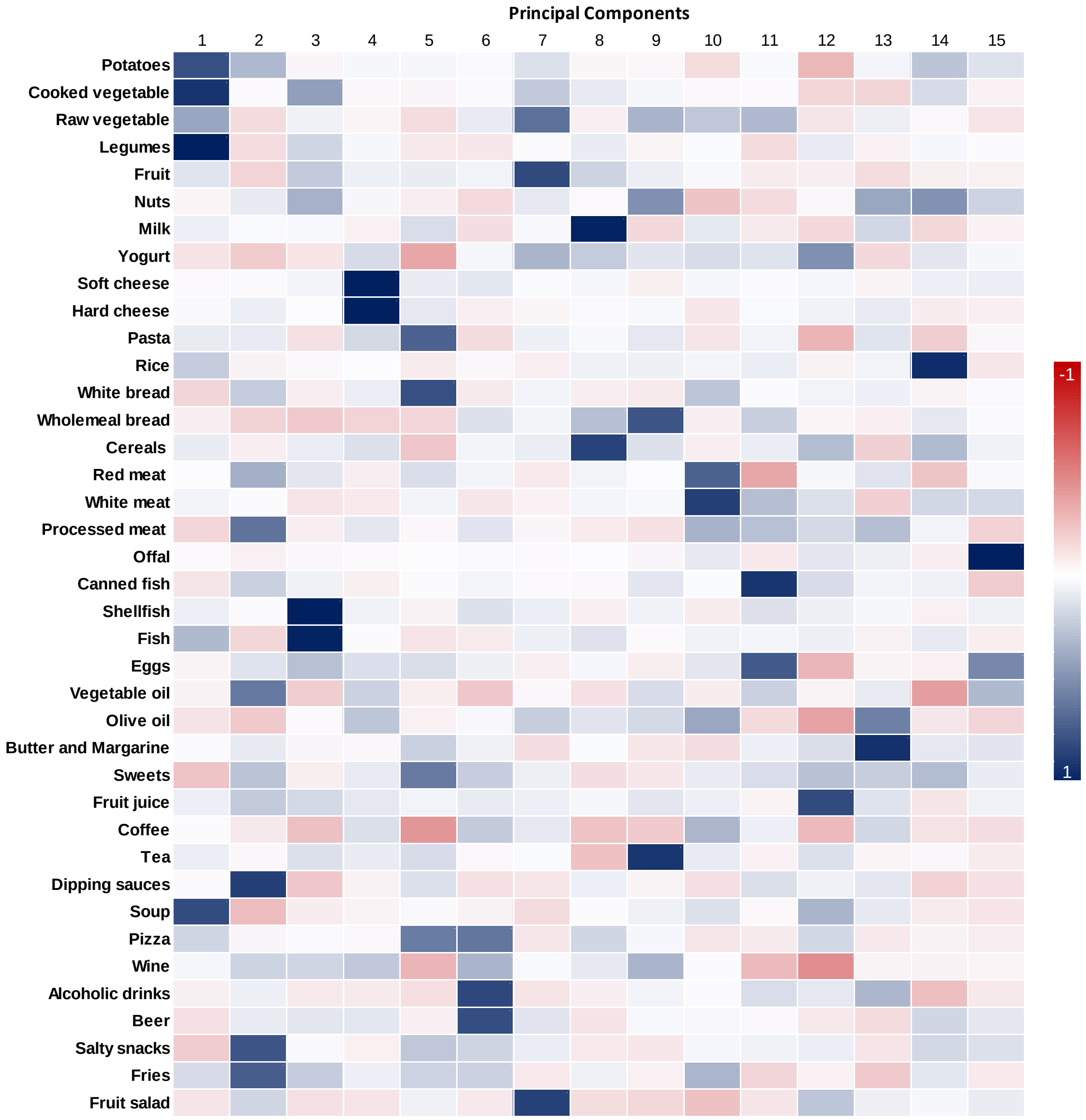

**Figure S2** Score plots of the first four principal components obtained after principal component analysis.

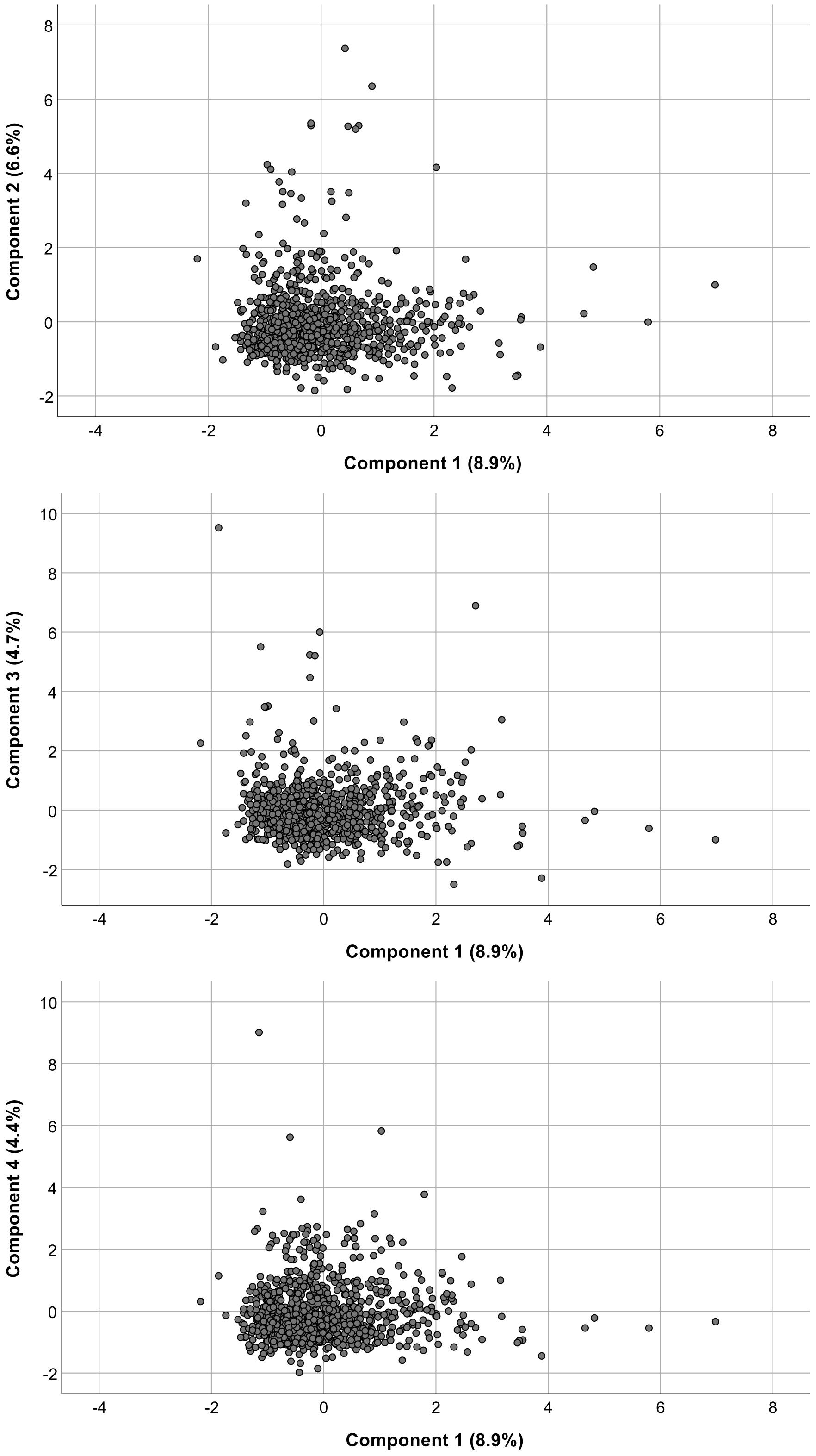

**Figure S3** Dendrogram of Hierarchical Clustering based on the Ward's criterion.

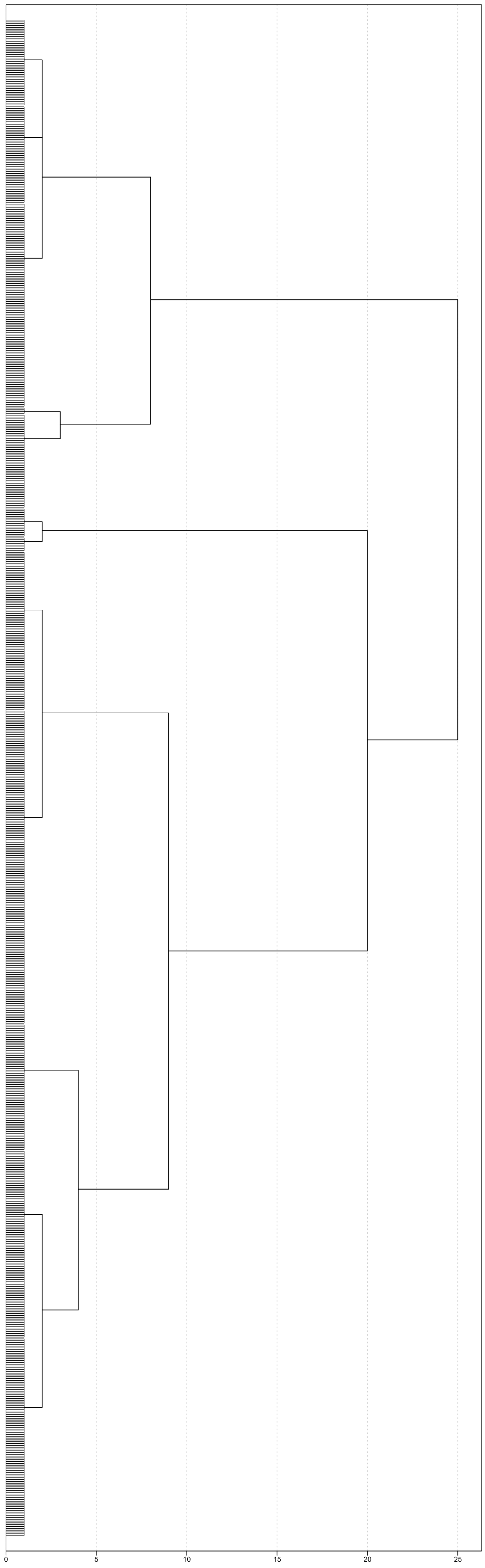

**Figure S4** Comparison of Silhouette score between different cluster solutions obtained through hierarchical clustering on principal components.

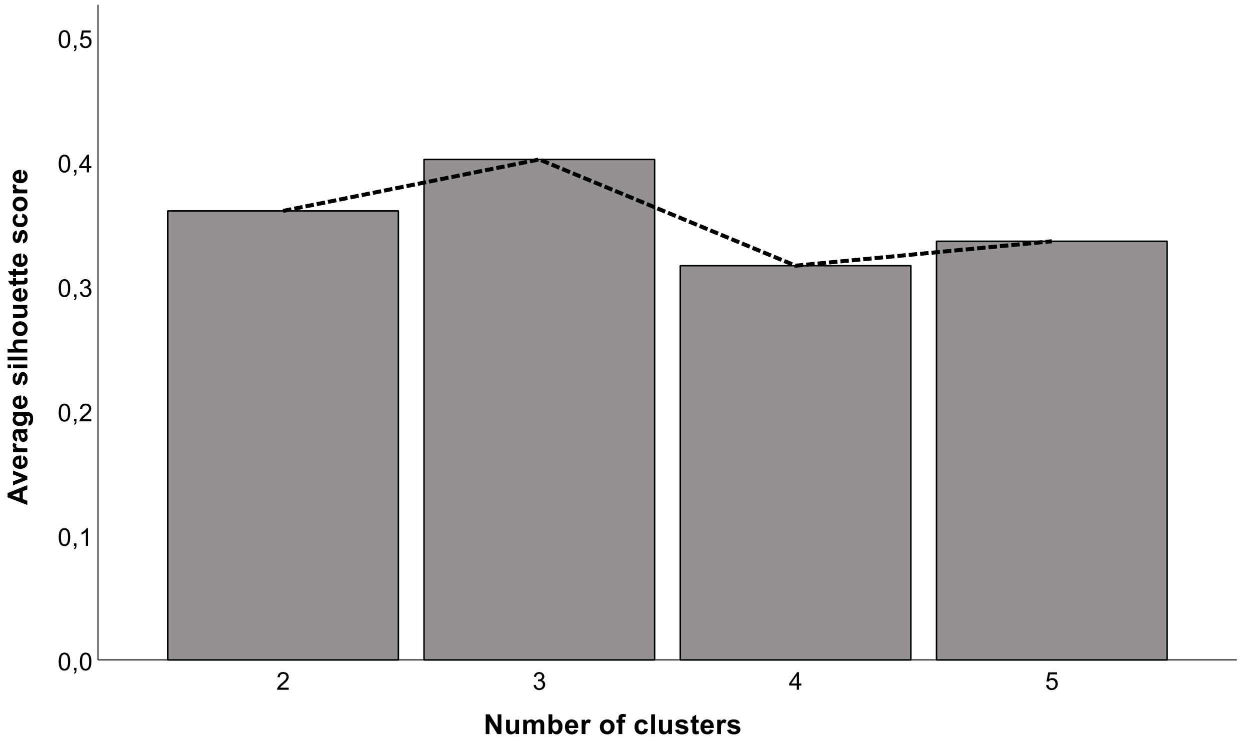

**Figure S5** Comparison of z-scores of food intakes between clusters obtained through clustering on four or on all principal components. Blue bars represent food categories that positively characterize the cluster. Red bars represent food categories that negatively characterize the cluster.

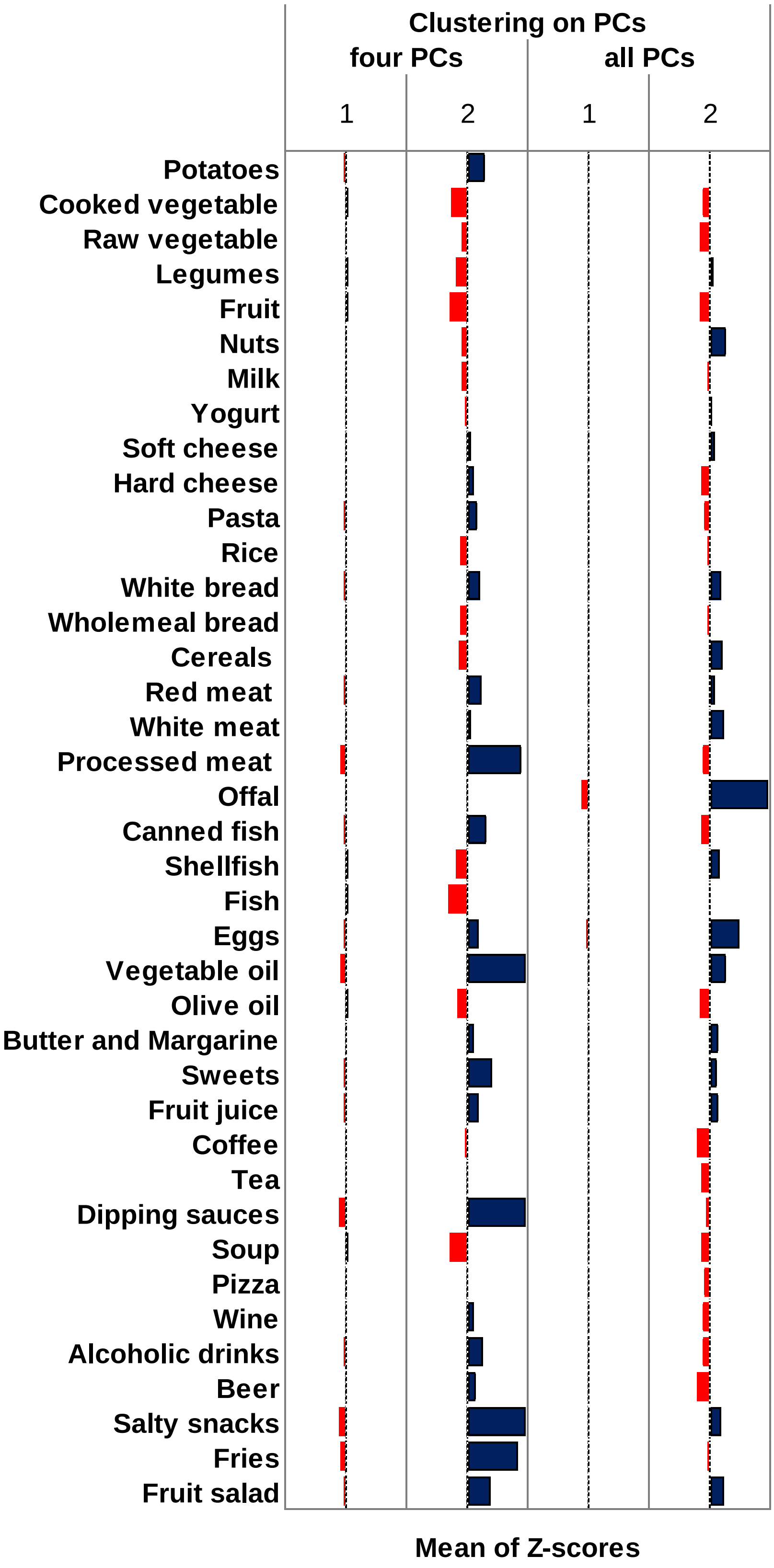

Supplement: Supplementary file 1 [file nutrients-15-00195-s001.zip › nutrients-2115830-supplementary.pdf]
